# EZH2 Regulates Protein Stability via Recruiting USP7 to Mediate Neuronal Gene Expression in Cancer Cells

**DOI:** 10.3389/fgene.2019.00422

**Published:** 2019-05-03

**Authors:** Anhua Lei, Lu Chen, Min Zhang, Xiaoli Yang, Liyang Xu, Ning Cao, Zan Zhang, Ying Cao

**Affiliations:** China’s Ministry of Education, Key Laboratory of Model Animals for Disease Study, Model Animal Research Center of Nanjing University, Nanjing, China

**Keywords:** cancer cell, neural progenitor cell, chromatin modification factors, gene regulation, neuronal differentiation, protein turnover, ubiquitination

## Abstract

Misexpression of chromatin modification factors and changed epigenetic modifications play crucial roles for tumorigenesis. Our previous studies demonstrated that inhibition of epigenetic modification enzymes EZH2, LSD1, DNMTs, and HDACs caused post-mitotic neuron-like differentiation in different cancer cells. However, how they regulate neuronal differentiation in cancer cells was unknown. Here, we show that EZH2, LSD1, DNMT1, and HDAC1 form interactions themselves, meanwhile, they also interact with SMAD proteins and β-CATENIN in cancer cells. Chemical inhibition of these enzymes leads to reduced level of proteins except HDAC1. The change in protein level and/or enzymatic activities further result in changed chromatin modifications on neuronal gene promoters, and activation of neuronal genes. Inhibition of these enzymes in neural progenitor cells (NPCs) also caused neuronal differentiation, similar to cancer cells. Particularly, EZH2 interacts with and required for the stability of LSD1, HDAC1, DNMT1, β-CATENIN, or SMAD2/4, via recruitment of deubiquitinase USP7. Reduced EZH2 leads to enhanced ubiquitination and degradation of these proteins, and decreased binding of LSD1, HDAC1, and DNMT1 to neuronal gene promoters, and lessened Wnt and TGFβ target gene activation. Hence, EZH2 sustains a series of proteins that promote tumorigenesis, in addition to its original function of histone methylation. Considering together with other studies, we conclude that these chromatin modification factors function in the same way in cancer cells as in neural progenitor/stem cells. The similarity between cancer cells and neural progenitor/stem cells provides an insight into the essence and unified framework for cancer initiation and progression, and are suggestive for novel strategies of cancer therapy.

## Introduction

Epigenetic modification factors are extensively involved in tumorigenesis due to their functions in chromatin modifications and consequently, in the regulation of gene transcription. DNA and histone methylation/demethylation and histone acetylation/deacetylation are among the major types of chromatin modifications. Enzymes responsible for these modifications are misregulatedduring tumorigenesis, leading to dysregulated epigenetics (Rodríguez-Paredes and Esteller, 2011; Dawson and Kouzarides, 2012; Suvà et al., 2013; Feinberg et al., 2016). Among chromatin modification enzymes, EZH2 is one of the best-known oncoproteins. It is the catalytic subunit of PRC2 complex and catalyzes trimethylation of histone H3 at Lys-27 (H3K27me3), causing transcription silencing. EZH2 is usually upregulated or its gene undergoes gain-of-function mutations in various solid cancer (Völkel et al., 2015; Kim and Roberts, 2016). Other pan-cancer promoting chromatin modification enzymes include LSD1/KDM1A, HDACs, DNMTs, etc. LSD1 demethylates histone H3 at Lys-4 and Lys-9; HDACs deacetylate lysine residues of core histones; whereas DNMTs catalyze methylation of CpG islands of DNA. These proteins all mediate transcriptional repression, are upregulated and overly active in most cancer types (Barneda-Zahonero and Parra, 2012; Subramaniam et al., 2014; Hosseini and Minucci, 2017).

Chromatin modification enzymes exhibit enzymatic specificity for different modification types, leading to transcriptional activation or silencing. Crosstalk has been noticed for some epigenetic modification factors and chromatin modifications. The crosstalk enables a concerted modification of chromatin, mediating a wide spectrum of cellular functions including chromatin remodeling, transcription, protein synthesis, signal transduction, and DNA repair (Biggar and Li, 2015). For example, crosstalk exists between LSD1 and HDACs in breast cancer cells. Combined inhibition of LSD1 and HDACs leads to an enhanced apoptosis and suppression of triple-negative breast cancer cells (Vasilatos et al., 2013). Besides dysregulated epigenetics in cancer cells, a few signaling pathways, e.g., Wnt/β-catenin and TGFβ signaling pathways, are hijacked by cancer cells and regulate every aspect of malignant properties. Some genes for key signal transducers, e.g., β-catenin and SMAD proteins, are frequently mutated or misregulated during tumorigenesis. Like crosstalk between different types of epigenetic modifications, the crosstalk between epigenetic modifications and these signaling pathways also exists, primarily through epigenetic regulation of the genes for signal transducers, as in the case of Wnt pathway (Ying and Tao, 2009).

These factors and pathways all play crucial roles in the regulation of cancer malignancy and upregulated in many different cancer types, and co-exist in cancer cells. Interestingly, our previously studies demonstrated that a major part of cancer promoting genes, including the genes for EZH2, LSD1, HDAC1/3, DNMT1, SMAD2/4, or β-catenin, are specifically expressed in embryonic neural cells (Zhang et al., 2017). Moreover, combined inhibition of EZH2, LSD1, HDACs, and DNMTs in cells of different cancer types led to post-mitotic neuron-like terminal differentiation, an effect resembling differentiation of neural progenitor/stem cells into post-mitotic neurons. This led us to a proposal that cancer cells resemble embryonic neural cells and that tumorigenesis represents a process of gradual loss of original cell identity and gain of properties of neural progenitor/stem cells (Cao, 2017; Zhang et al., 2017). Co-existence of these factors in a same cellular context suggest that interactions between these factors might occur in cancer cells to achieve a coordinated state of different chromatin modifications or signaling pathways, a state being required for cancer cell physiological functions, e.g., expression of genes mediating differentiation of cancer cells. Nevertheless, whether this is true remained a topic to elucidate.

Here, we show the interactions between the epigenetic modification enzymes and between the enzymes and SMAD2/4 and β-Catenin. Chemical inhibition of these enzymes causes regulatory effect of their interaction partners except HDAC1, changed protein-promoter binding, and changed chromatin modifications on the promoters of neuronal differentiation genes. We also show that EZH2 plays a key role in the maintenance of stability of its interaction partners, via interaction with deubiquitase USP7, so as to maintain a network promoting cancer initiation and progression. Importantly, inhibition of these enzymes together leads to neuronal differentiation in neural progenitor/stem cells, strengthening the similarity between cancer cells and neural progenitor/stem cells.

## Materials and Methods

### Cell Culture

The hepatocellular carcinoma cell line HepG2 and HEK293T cells were cultured in Dulbecco’s modified eagle medium (DMEM. Thermo Fisher Scientific, #11965-092), and the colon adenocarcinoma cell line SW480 was cultured in Leibovitz’s L-15 medium (L-15. Thermo Fisher Scientific, #41300039). All culture media were supplemented with 10% fetal bovine serum (FBS. Hyclone, #SH30084.03) and with 50 U/ml penicillin and 50 μg/ml streptomycin. All cells were grown at 37°C with 5% CO_2_. Cells were obtained from the Cellbank of the Shanghai Institutes for Biological Sciences (Shanghai, China).

Mouse embryonic stem cells (mESCs) were cultured in DMEM medium supplemented with 15% FBS, 1 ng/ml human LIF (CST. #8911), 100 μM β-mercaptoethanol, 2 mM L-glutamine (Thermo Fisher Scientific, #25030164), 1× MEM nonessential amino acids (Thermo Fisher Scientific, #11140050), and penicillin/streptomycin. Neural progenitor cells (NPCs) were derived from mESCs by incubating mESCs in serum-free neural differentiation medium Ndiff 227 (CellArtis, #Y40002).

### Protein Co-immunoprecipitation

Anti-rabbit or mouse IgG, or different primary antibodies against endogenous proteins were linked to protein G-sepharose (Amersham, #17-0618-02) by incubating an antibody with sepharose beads in PBS. After being washed, the IgG or specific antibody-conjugated beads were added to cellular extracts and incubated overnight at 4°C. Beads were then washed in TBST buffer (pH7.2, 25 mM Tris-HCl, 150 mM NaCl, 0.5% Tween-20). Immuno-complexes were eluted by incubating the beads in 1× loading buffer at 95°C, and subjected to SDS-PAGE.

### Time-Course Treatment of Cells With Cycloheximide

HepG2 cells were infected with lentivirus containing shEZH2 and selected by puromycin selection. When growing to 80–90% confluency, cells were treated with cycloheximide (CHX) at a final concentration of 50 μg/ml for 0, 2, 4, 6, and 8 h, respectively. A control experiment was performed in parallel by infecting HepG2 cells with virus containing only the empty vector for short-hairpin construct (shCtrl). Cells were collected and subjected to immunoblotting.

### Immunoblotting (IB) and Quantification of Protein Levels Detected by IB

IB with whole cell lysates, nuclear extracts or immunoprecipitated eluates was performed using conventional SDS-PAGE. Blots were detected with a Western blotting substrate (Tanon, #180-501). The primary antibodies were (fold of dilutions indicated): βACT (CST, #8480. 1:2,000), CCND1 (CST, #2978. 1:2,000), DNMT1 (Abcam, #ab13537. 1:2,000), ERBB2 (CST, #4290. 1:2,000), EZH2 (CST, #5246. 1:4,000), ETS1 (CST, #14069. 1:2,000), FAK (CST, #3285. 1:2,000), FOXM1 (CST, #5436. 1:2,000), GAPDH (Santa Cruz, #sc-25778. 1:2,000), HDAC1 (CST, #5356. 1:2,000), HES1 (CST, #11988. 1:2,000), LSD1 (CST, #2139. 1:2,000), β-CAT (CST, #8814. 1:2,000), MAP2 (CST, #8707. 1:2,000), NEUROD1 (CST, #4373. 1:2,000), NFM (CST, #2838. 1:2,000), OCT4 (CST, #2750. 1:2,000), SNAIL (CST, #3879. 1:2,000), SLUG (CST, #9585. 1:2,000), p-STAT3 (CST, #9145. 1:2,000), pSMAD1 (CST, #9511. 1:2,000. For detecting the activated SMAD1 phosphorylated at Ser463/465), pSMAD2 (CST, #3101. 1:2,000. For detecting the activated SMAD2 phosphorylated at Ser465/467), SMAD2/3 (CST, #5678. 1:2,000), SMAD4 (CST, #9515. 1:2,000), SUZ12 (CST, #3737. 1:2,000), SYN1 (CST, #5297. 1:2,000), TBP (Santa Cruz, #sc-204. 1:1,000), SETD1A (Bethyl Laboratories, #A300-289A-M. 1:2,000), USP7 (CST, #4833. 1:2,000), and YAP (CST, #8418. 1:2,000).

In experiments detecting the effect of EZH2 knockdown on protein level or the dependence on proteosomal degradation and in experiments detecting the effect of EZH2 knockdown on protein half-lives, protein levels were quantified with ImageJ 1.48 software. The expression levels of each protein were normalized to the levels of β-ACT, and then compared with expression levels in control cell (shCtrl) or cells without CHX treatment (0 h), both of which were set to 1. Experiments were carried out in triplicate and results were shown as the mean ± SEM.

### Chemical Treatment of Cancer Cells and Neural Progenitor Cells

To achieve neuron-like differentiation effect, treatment of HepG2 cells with specific chemical inhibitor for DNMTs (5-aza-2′-deoxycytidine. AZA) (Selleckchem, #S1200), EZH2 (EPZ-6438. Selleckchem, #S7128), LSD1 (SP2509. Selleckchem, #S7680) and class I HDACs (TSA. Selleckchem, #S1045) was performed exactly as described (Zhang et al., 2017). Induction of neuronal differentiation from NPCs was as follows: after mESCs had been transferred from stem cell culture medium to Ndiff 227 medium for 2 days, TSA, EZH2 inhibitor EPZ-6438 and LSD1 inhibitor SP2509 were first added to Ndiff 227 medium at a final concentration of 5 nM each. AZA was added 2 days later, also at a final concentration of 5 nM. Cells were cultured until neurite outgrowth began. Afterward, control cells treated with DMSO and differentiated cells were subjected to additional analyses.

### Knockdown and Overexpression Constructs

A short-hairpin RNA (shRNA)-based functional knockdown approach was used to investigate the effect of genes of interest on cells via lentiviral infection. shRNAs for functional knockdown of *DNMT1* (shDNMT1), *EZH2* (shEZH2), *HDAC1* (shHDAC1), *HDAC3* (shHDAC3), *LSD1* (shLSD1) were as described (Zhang et al., 2017). shRNA for human *USP7* (shUSP7) was a validated MISSION^®^ shRNA TRCN0000004058 (Sigma-Aldrich), which was cloned to pLKO.1 vector. The empty pLKO.1 vector was used as a control (shCtrl).

The coding regions of *Xenopus laevis ezh2a* (AF351126) and *usp7* (XM_018237116) were subcloned to pCS2+6 × MTmcs or pCS2+4 × HAmcs vector to make fusion constructs used for transient overexpression in cells.

Plasmids for tagged Ezh2 or Usp7 was transfected to HEK293T, HepG2 or SW480 cells using PEI. Seventy-two hours after transfection, cells were subjected to immunofluorescence (IF) assays. SB431542 (Sigma-Aldrich, #S4317) was used at a final concentration of 10 μM to treat cells for 16 hrs before cell collection and IF or Western blotting assays.

### Viral Infection of Cells

For stable knockdown assays, virus packaging plasmids and pLKO.1 empty vector plasmid that was used as control, or constructs containing shRNAs against different genes were transfected into HEK293T cells using polyethylenimine (PEI). Forty-eight hours after transfection, polybrene at a final concentration of 10 μg/ml was added to the lentiviral supernatant. The supernatant was then filtered through a 0.45 μm filter and used for infecting cells. Forty-eight hours after infection, cells were selected with puromycin at 2 μg/ml in culture for 2 days, and cultured further until significant phenotype was observed (for detecting the effect of knockdown on cancer cell line differentiation) or harvest for additional analyses.

### Immunofluorescence

Neurospheres for neuronal differentiation, or HEK293T cells with transient overexpression were cultured on coverslips in 6-well plates. Afterward, cells were washed with phosphate buffered saline (PBS) thrice, fixed with 4% PFA for 15 min, which was inactivated with 50 mM ammonium chloride in PBS for 10 min. Cells were then permeabilized with 0.1% Triton X-100 for 10 min, blocked with 0.2% fish skin gelatin (Sigma-Aldrich, #G7041) for 30 min at room temperature. Subsequently, cells were incubated with primary antibodies against SOX1 (Abcam, #ab87775. 1:500), PAX3 (Abcam, # ab15717. 1:200), MAP2 (CST, #8707. 1:200), TUBB3 (CST, #5568. 1:200), HA-tag (CST, #2367. 1:500), Myc-tag (Sigma, #C3956. 1:500), nonP-β-CAT (CST, #8814. 1:500), LSD1 (CST, #2139. 1:500), SMAD2 (CST, #5678. 1:500), SMAD4 (CST, #9515. 1:500), DNMT1 (CST, #5032. 1:500), HDAC1 (CST, #5356) at 4°C overnight. The secondary antibody was Cy3-conjugated anti-rabbit IgG (Sigma-Aldrich, #C2306. 1:1,000), anti-mouse IgG (FITC-conjugated) (Sigma-Aldrich, #F9137. 1:1,000), and Alexa Fluor^®^568 donkey anti-Rabbit IgG (H+L) (Invitrogen, #A10042. 1:1,000). Cells were counterstained with DAPI to view cell nuclei. After being rinsed, coverslips were mounted with anti-fade mounting medium (Invitrogen, #S36936). Cells were then detected using fluorescence microscope (FluoView FV1000, Olympus, Leica TCS SP5 II).

### Cellular Extract Preparations

Whole cell lysates were used for detecting protein level in cells. Cells were washed with ice-cold PBS and lysed on ice for 40 min in lysis buffer containing 150 mM NaCl, 0.5% NP-40, 0.25% sodium deoxycholate, 50 mM Tris (pH 8.0), protease inhibitor cocktail (Roche. #04693132001) and phosphatase inhibitor cocktail (Roche. #04906845001). Lysates were cleared via centrifugation.

For preparation of cellular nuclear extracts, cultured cells were trypsinized and washed twice with PBS. Cells were re-suspended in 5 times of packed cell volume (PCV) of hypotonic buffer supplemented with protease inhibitor cocktail (Roche. #04693132001), centrifuged, and suspended again in 3× PCV of hypotonic buffer. Cells were lysed for 10 min on ice with shaking, and cell nuclei were precipitated. Nuclei were washed with PBS, and lysed in buffer (pH 7.5, 50 mM Tris-HCl, 150 mM NaCl, 5 mM EDTA 0.5% NP-40 Substitute) supplemented with protease inhibitor cocktail on ice for 20 min, and spun down to discard cell debris.

### Gene Expression Profiling Analysis on SW480 Cells in Response to EZH2 Knockdown

Gene expression of SW480 cells infected with lentivirus carrying the empty vector (shCtrl) or carrying shEZH2 (shEZH2) was examined using gene expression microarray. After significant phenotypic change occurred in cells with EZH2 knockdown, both control and knockdown cells were collected. RNA extraction/purification, cRNA probe synthesis, probe hybridization to microarrays, signal processing, raw data analysis, and the subsequent enrichment and annotation of pathway, disease ontology and gene ontology were as exactly described (Zhang et al., 2017). Results are shown as bar charts. Raw data can be found in GEO under accession number GSE118593^1^.

A heat map for differentially expressed neuron enriched genes, which were extracted from the differentially expressed genes (DEGs) in the “Biological Process,” were generated using Gene Cluster 3.0 and TreeView.

### Chromatin Immunoprecipitation (ChIP)

ChIP was performed with HepG2 cells treated vehicle or chemical inhibitor combination TALE according to conventional methods with minor modifications. Briefly, fresh formaldehyde solution was added to the culture medium in a culture plate to a final concentration at 1% to crosslink protein-DNA complexes for 10 min at room temperature. Crosslinking was quenched for 5 min by adding 1/20 volume of 2.5 M glycine. Cells were then washed twice with ice-cold 1× PBS, trypsinizated, washed again with PBS. After centrifugation, cells were lysed for 20 min on ice with 1 ml of hypotonic buffer (10 mM HEPES pH7.9, 1.5 mM MgCl_2_, 10 mM KCl, 0.5 mM DTT) plus protease inhibitor cocktail (Roche. #04693132001). Lysates were pelleted via centrifugation at 3,000 rpm for 5 min at 4°C, followed by re-suspension of pelleted cell nuclei in nuclear lysis buffer (1% SDS, 10 mM EDTA, 50 mM Tris-HCl pH8.0) supplemented with protease inhibitors. After nuclear lysis for 20 min at 4°C, ChIP dilution buffer (0.01% SDS, 1% Triton X-100, 2 mM EDTA, 20 mM Tris-HCl pH 8.0), 150 mM NaCl, plus protease inhibitors) was added to nuclear lysates. Samples were then sonicated with a sonicator (Bioruptor^TM^ USD-200) for 10 cycles of 30 s pulse followed by 30 s rest at HIGH power output. After centrifugation, the supernatants containing sheared chromatin were pre-cleared with protein-G agarose that was pre-blocked with 1% BSA in PBS. 50 μl of pre-cleared chromatin was used as input control. The remaining part of each sample (DMSO and TALE) was divided into equal parts, each part being incubated with 5 μg of antibody against DNMT1, EZH2, LSD1, HDAC1, and rabbit or mouse IgG (depending on host species of an antibody) overnight at 4°C. Similar experiments were performed by incubating pre-cleared chromatin with antibodies against different histone modifications H3K4me1, H3K27me3, H3K27ac, H3K9ac, and IgG. Afterward, immunocomplexes were collected by precipitation with pre-blocked protein-G agarose, followed by washing agarose beads thrice with low salt buffer (1% Triton X-100, 2 mM EDTA, 50 mM HEPES pH 7.5, 150 mM NaCl) and once with TE buffer. DNA was eluted from agarose beads with reversal of cross-linking and proteinase K digestion, extracted and precipitated with conventional phenol-chloroform-ethanol strategy. A series of primer pairs were designed to amplify different regions in promoters of *CDKN1A, CDH1, NEUROD1*, and *TUBB3* using quantitative PCR (qPCR) on an ABI 7300 system. Amplification parameters were as follows: one cycle of pre-denaturation at 95°C for 5 min, followed by 40 cycles of denaturation at 95°C for 10 s, annealing and extension at 60°C for 30 s, and an additional cycle for melting curve. Cross-points were calculated using ABI 7300 system SDS software. After normalized with levels of chromatin precipitated with IgG, changes in protein-DNA binding or changes in histone/DNA modifications in the detected promoters were calculated by comparing the levels of precipitated chromatin by a specific antibody from TALE treated or EZH2 knockdown cells and from control cells (DMSO or shCtrl). Experiments were carried out in triplicate. Significance in changes of levels of precipitated chromatin by a specific antibody was calculated using unpaired Student’s *t*-test. Final results were presented as histograms with relative units. Primers for qPCR are listed in Supplementary Table 1.

### Quantitative Reverse Transcriptase-Polymerase Chain Reaction (qRT-PCR)

Total RNAs were extracted from control or treated cells with TRIzol reagent (Invitrogen). cDNAs were transcribed from total RNAs using the HiScript II 1st Strand cDNA Synthesis Kit (+gDNA wiper) (Vazyme, #R212-01/02) which contains reagent removing genomic DNA. qPCR was performed in the same way as used for ChIP assays. Experiments were performed in triplicate. Significance in changes of gene expression was calculated using unpaired Student’s *t*-test. Data were shown as histograms with relative units, with expression level in control being set to 1. Expression of *GAPDH* was used as a loading control. Primers for each detected gene are listed in Supplementary Table 2.

### Luciferase Assay

The SMAD-binding element (SBE) luciferase reporter SBE4-Luc (Addgene, #16495) and TopFlash was used for detecting the effect EZH2 knockdown on target gene transcription of TGFβ and Wnt signaling, respectively. HepG2 cells were first infected with lentivirus carrying shEZH2 or empty vector (shCtrl) and selected with puromycin at 1 μg/ml for 2 days. Afterward, when cells were grown at ∼80% confluency, 300 ng of SBE4-Luc reporter and 1 ng of Renilla luciferase reporter plasmids were co-transfected to HepG2 cells for 48 h. In parallel, an empty vector pGL3-basic (Promega) and was also transfected in the same way. Similarly, the Wnt-responsive reporter TopFlash and its negative control FopFlash were transfected to HepG2 cells, for monitoring Wnt target transcription after knockdown. Luciferase activity was measured using the Dual-Luciferase Assay System (Promega, #E1960). Data are shown as the mean ± SEM of SBE4-Luc/vector or TopFlash/FopFlash ratios that were obtained from at least three independent experiments. Significance was calculated using Student’s *t*-test.

## Results

### Epigenetic Modification Enzymes and Wnt or TGFβ Signal Transducers Form Protein Interactions, Working Cooperatively to Regulate Gene and Protein

**FIGURE 1:**
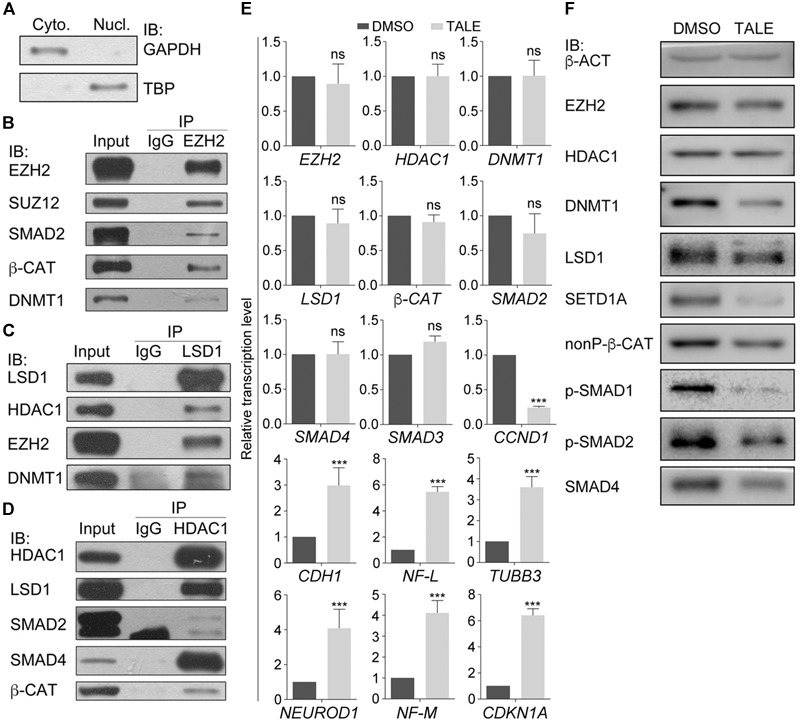
Protein interactions in cancer cell lines and differential effect of combined chemical inhibition on gene expression and protein. **(A–D)** Chromatin modification enzymes and signal transducers forms interactions in HepG2 cell nuclei. **(A)** Immunoblotting (IB) detection of the effect of nuclear protein extraction. GAPDH was used as a marker for cytosolic fraction (Cyto.) and TBP was used as a marker for nuclear fraction (Nucl.). **(B–D)** Different proteins were detected from the immunoprecipitates that were precipitated from the nuclear lysates with the antibody against EZH2 **(B)**, LSD1 **(C)**, and HDAC1 **(D)**. In each experiment in **(B–D)**, co-IP with an IgG antibody was performed in parallel as a negative control, and the protein level in the nuclear lysates was used as positive control (Input). **(E)** TALE treatment caused different regulatory effect on transcription of genes, as examined with qRT-PCR. Significance in gene expression change was calculated for experiments in triplicate using Student’s *t*-test. Data are shown as mean ± SEM. ns, not significant. ^∗∗∗^*p* < 0.001. **(F)** Immunoblotting detection of protein level in cell without (DMSO) and with TALE treatment. β-ACT was used as a loading control.

Our previous work (Zhang et al., 2017) demonstrated that treatment simultaneously with HDAC inhibitor TSA (T), DNMT inhibitor AZA (A), LSD1 inhibitor SP2508 (L), and EZH2 inhibitor EPZ-6438 (E) led to post-mitotic neuron-like differentiation, whereas treatment with an individual inhibitor usually resulted in weaker or no significant changes in cancer cells, suggesting that these epigenetic modification enzymes may work cooperatively to regulate the neuron-like differentiation effect in cancer cells. We also observed a significant downregulation of SMAD proteins and β-CAT, the key signal transducers of TGFβ and Wnt signaling pathways. Co-immunoprecipitation (co-IP) assays with nuclear extracts from hepatocellular carcinoma cell line HepG2 (Figures 1A–D) were performed. Nuclear extracts were confirmed by the expression of the nuclear protein TBP in the nuclear fraction of cellular extracts and expression of nonnuclear protein GAPDH in the cytosolic fraction (Figure 1A). Co-IP with HepG2 cells (Figures 1B–D) demonstrated that, in addition to the known EZH2 interaction partner SUZ12, SMAD2, β-CAT, and DNMT1 were also precipitated by an EZH2 antibody (Figure 1B), suggesting that EZH2 can form complexes with SMAD2, β-CAT, and DNMT1. Moreover, the LSD1 antibody was able to precipitate EZH2 and DNMT1, in addition to its characterized interaction partner HDAC1 (Figure 1C), and the HDAC1 antibody could precipitate SMAD2, SMAD4, and β-CAT (Figure 1D).

We asked whether these proteins or their genes were regulated in response to combined inhibition of EZH2, LSD1, DNMT1, and HDAC1. TALE inhibitors didn’t affect transcription of *EZH2, LSD1, DNMT1, HDAC1, β-CAT, SMAD2*, or *SMAD4* in HepG2 cells (Figure 1E), but downregulated *CCND1*, and upregulated *CDKN1A* and neuron-specific genes *NF-L, NF-M, NEUROD1*, and *TUBB3* (Figure 1E). *CDH1* was also upregulated in treated cells. *CDH1* has always been used as a marker gene for epithelial cells. However, it mediates neurite outgrowth during neuronal differentiation (Oblander et al., 2007; Oblander and Brady-Kalnay, 2010). This is in agreement with the neuronal differentiation effect after TALE treatment. In contrast to unaltered transcription, protein level of EZH2, LSD1, DNMT1, β-CAT (nonP-β-CAT, the nonphosphorylated, active form of β-CAT), SMAD2 or SMAD4 was all reduced after TALE treatment, except that HDAC1 was not changed significantly (Figure 1F). Additionally, SETD1A, an epigenetic modification enzyme that specifically methylates histone H3 at Lys 4, was also reduced (Figure 1F). The results suggest that there exists crosstalk between these epigenetic modification enzymes, and crosstalk between the enzymes and signal transducers, thereby mediating expression of genes that regulate cancer cell malignancy and neuronal differentiation.

### Binding of Epigenetic Modification Enzymes to Promoters and Chromatin Modifications in the Promoters Are Changed in Cells Treated With TALE

**FIGURE 2:**
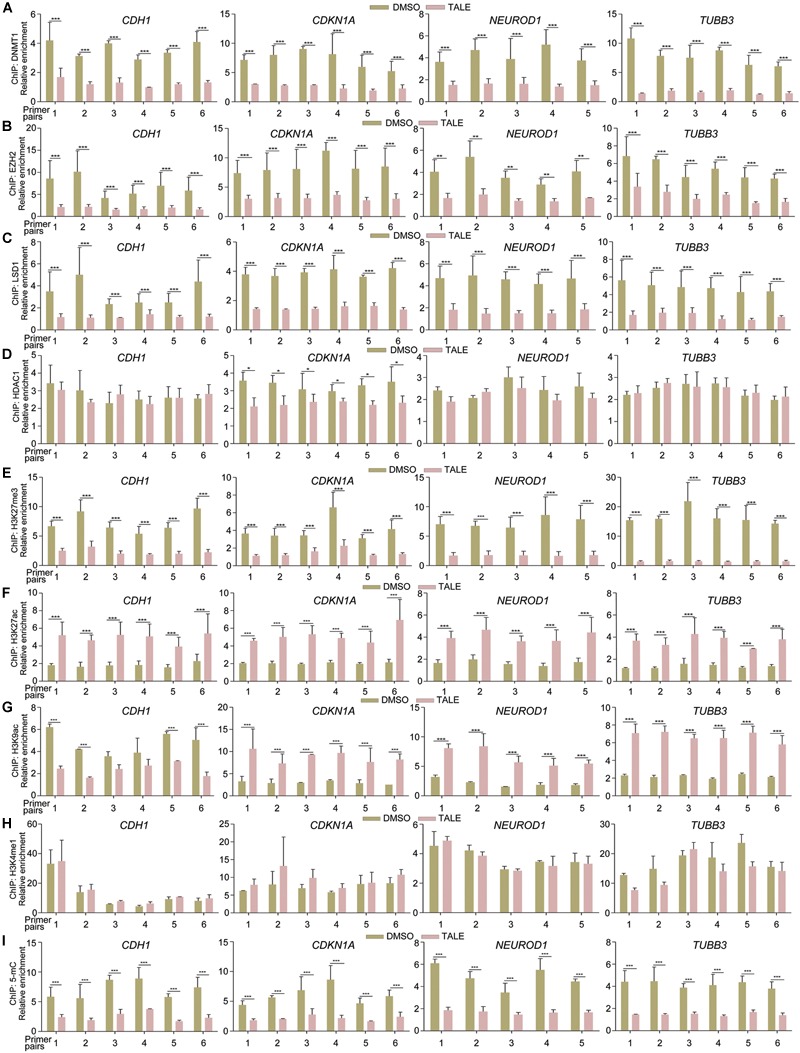
TALE treatment affects the binding of epigenetic modification enzymes to and alters chromatin modifications in gene promoters. **(A–D)** Chromatin immunoprecipitation (ChIP) assays of binding of DNMT1 **(A)**, EZH2 **(B)**, LSD1 **(C)**, and HDAC1 **(D)** to promoter regions of *CDH1, CDKN1A, NEUROD1*, and *TUBB3* in HepG2 cells that were treated with DMSO or TALE. **(E–I)** ChIP assays of changes of histone modifications H3K27me3 **(E)**, H3K27ac **(F)**, H3K9ac **(G)**, H3K4me1 **(H)**, and DNA methylation 5-mC **(I)** in promoters of *CDH1, CDKN1A, NEUROD1*, and *TUBB3* in HepG2 cells treated with DMSO or TALE. In each panel, the numbers of the vertical axis are arbitrary units indicating relative enrichment of chromatin fragments precipitated by an antibody; the numbers of the horizontal axis represent different amplified regions of a promoter, as described in Materials and Methods. Significance of changes in enrichment of chromatin fragments from cells with or without TALE treatment was calculated for experiments in triplicate using Student’s *t*-test. Data are shown as mean ± SEM. ^∗^*p* < 0.05, ^∗∗^*p* < 0.01, ^∗∗∗^*p* < 0.001.

Due to transcriptional upregulation of *CDH1, CDKN1A, NEUROD1*, and *TUBB3*, we tested whether epigenetic modification enzymes regulate these genes directly. Chromatin immunoprecipitation (ChIP) assays displayed that antibodies against DNMT1, EZH2, or LSD1 were able to precipitate promoter fragments of *CDH1, CDKN1A, NEUROD1*, and *TUBB3* (Figures 2A–C), and the precipitated promoter fragments were strongly reduced in cells with TALE treatment (Figures 2A–C). Therefore, DNMT1, EZH2, or LSD1 bind to the promoters of these genes, thereby regulating their transcription. In contrast, HDAC1 showed no significant changes in binding to promoters of *CDH1, NEUROD1*, and *TUBB3*, and slightly decreased binding to *CDKN1A* promoter regions (Figure 2D), in agreement with nearly unchanged HDAC1 expression after TALE treatment.

We further explored whether decreased activity of EZH2, DNMT1, LSD1, or HDAC1 would alter modifications on these promoters. TALE treatment in HepG2 cells resulted in strongly decreased tri-methylation of histone H3 at Lys 27 (H3K27me3), a well-characterized transcriptional repression mark, on promoter regions of *CDH1, CDKN1A, NEUROD1*, and *TUBB3* (Figure 2E). Acetylation of H3 at Lys 27 (H3K27ac), a transcriptional activation mark, was increased significantly on these promoters (Figure 2F). Another transcriptional activation mark, acetylation of histone H3 at Lys 9 (H3K9ac), showed different patterns of change. It was increased in *CDKN1A, NEUROD1*, and *TUBB3* promoters in response to TALE treatment (Figure 2G). However, there was a tendency of decrease in H3K9ac modification in promoter regions of *CDH1* (Figure 2G). The general tendency in increase of histone acetylation modifications on these promoters is a result of the inhibition of HDAC enzymatic activity. Mono-methylation of histone H3 at Lys 4 (H3K4me1) also marks transcriptional activation. This mark showed no significant change on promoters of *CDH1, CDKN1A, NEUROD1*, and *TUBB3* (Figure 2H). That decreased LSD1 binding did not cause an increased H3K4me1 modification on these promoters should be due to a lack of active methylation at this site, resulting from reduced SETD1A (see Figure 1F and also text below). Besides these histone marks, we also observed decreased levels of 5-methylcytosine (5-mC), which marks transcriptional repression state, on these promoter regions (Figure 2I). Hence, there is a general tendency of decrease in H3K27me3 and 5-mC, and a tendency of increase in H3K27ac and H3K9ac in *CDH1, CDKN1A, NEUROD1*, and *TUBB3* promoters. This is in agreement with decreased expression and activity of epigenetic modification enzymes, and explains well the transcriptional activation effect on neuronal differentiation genes after TALE treatment.

### TALE Treatment Leads to Neuronal Differentiation in Neural Progenitor Cells

**FIGURE 3:**
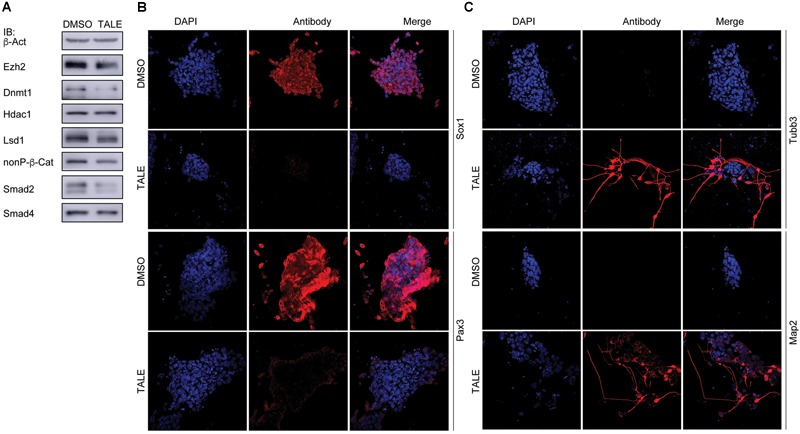
TALE treatment of neural progenitor cells causes neuronal differentiation. **(A)** IB detection of protein level of the epigenetic modification enzymes in neurospheres without (DMSO) and with TALE (TALE) treatment. β-Act: loading control. **(B)** Immunofluorescence (IF) detection of the markers for neural stemness, Sox1 and Pax3, in neurospheres without and with TALE treatment. **(C)** IF detection of the markers for neuron cells, Tubb3 and Map2, in neurospheres without and with TALE treatment. In **(B,C)**, cell nuclei were counterstained with DAPI.

Post-mitotic neuron-like differentiation effect in various cancer cell lines after TALE treatment raised the question whether TALE could also induce neuronal differentiation in NPCs. We made use of mouse embryonic stem cell (mES)-derived NPCs, because ES cells assume a neural progenitor/stem cell fate in serum-free culture by the neural default pathway (Smukler et al., 2006). As observed in cancer cells, TALE treatment also caused a decreased expression of Ezh2, Lsd1, Dnmt1, β-catenin, and Smad proteins in NPCs, but not a decreased Hdac1 (Figure 3A). NPCs without TALE treatment displayed strong immunofluorescence (IF) staining for the markers of neural progenitor/stem cells, Sox1 and Pax3, indicating neural stemness of these cells, however, staining for these markers almost vanished in TALE treated cells, showing the loss of neural stemness (Figure 3B). By contrast, control NPCs showed no staining for markers of neuron cells, Tubb3 and Map2; whereas TALE treatment caused strong staining for these neuron markers, an indication of neuronal differentiation (Figure 3C). Therefore, by inhibition of a same set of epigenetic modification enzymes, NPCs and cancer cells undergo similar neuronal differentiation, reinforcing that cancer cells are analogous to neural progenitor/stem cells.

### Knockdown of EZH2, but Not Other Epigenetic Modification Factors, Leads to Neuron-Like Differentiation in SW480 Cells and Repression of Cancer-Promoting Proteins

**FIGURE 4:**
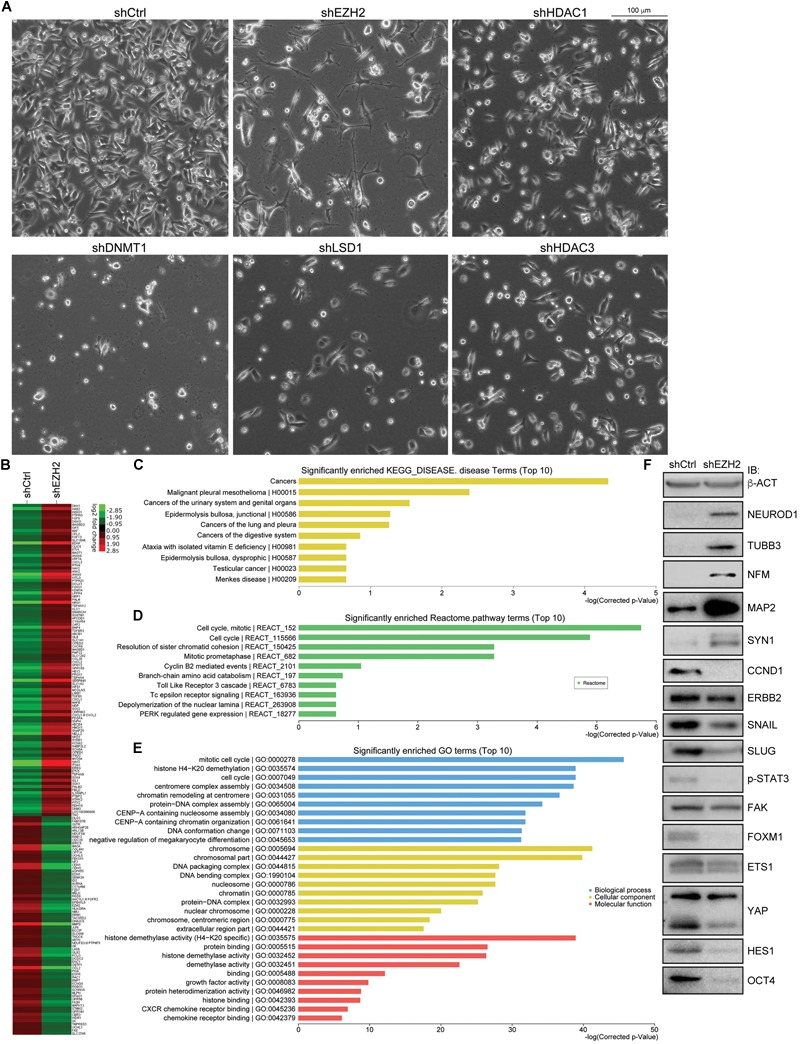
EZH2 knockdown induces neuronal differentiation in SW480 cells and represses signals promoting malignancy. **(A)** Knockdown of EZH2 generated a significant neuronal phenotype in SW480 cells, as compared with knockdown either of HDAC1, HDAC3, LSD1, or of DNMT1. **(B)** Heatmap based on a gene expression profiling assay showing expression changes of neuronal genes in cells without (shCtrl) or with EZH2 knockdown (shEZH2). **(C–E)** The enriched disease terms **(C)**, Reactome pathway terms **(D)** and GO terms **(E)** for differentially expressed genes from the profiling assay. **(F)** IB assay showing the effect of EZH2 knockdown on the expression of neuron-specific proteins and proteins that are involved in promoting malignanacy. β-ACT was used as a loading control.

Single knockdown of HDAC1, HDAC2, HDAC3, LSD1, DNMT1, and EZH2 generated different effects of neuron-like differentiation in cell lines of different cancer types (Zhang et al., 2017). It seems that EZH2 knockdown exhibits stronger neuron differentiation effect in cancer cell lines. In particular, knockdown of EZH2, but not other enzymes, caused strong neuron-like differentiation in colorectal cell line SW480 cells (Figure 4A). Knockdown of DNMT1 caused cell death during culture, and knockdown of HDAC1, HDAC3, or LSD1 did not generate significant morphological alteration (Figure 4A). A gene expression profiling assay showed that EZH2 knockdown in SW480 cells led to an upregulation of 100 neuron-enriched genes, in contrast to downregulation of 70 genes (Figure 4B), supporting the gain of neuronal phenotype. Among all the 691 DEGs after EZH2 knockdown, the most enriched disease genes are related to cancer in general, followed by some specific types of cancer (Figure 4C), implying that EZH2 is a master regulator of cancer related genes. Moreover, the most enriched Reactome pathway terms are associated with cell cycle (Figure 4D). The most enriched gene ontology (GO) terms for biological process are also associated with cell cycle (Figure 4E). This is reasonable in that both, cancer cells and neural progenitor/stem cells, are highly proliferative. Neuronal differentiation causes cells to exit from cell cycles. The most enriched GO terms for cellular component are associated with chromosome, and those for molecular function are associated with histone demethylase activity (Figure 4E), in agreement with functions of EZH2 in chromatin modification. We also detected the expression of proteins that are involved in neuronal differentiation and cancer initiation and progression. The result confirmed that NEUROD1, TUBB3, NFM, MAP2, and SYN1, which are typical neuronal proteins, were strongly upregulated (Figure 4F). In contrast, proteins that promote various cancer malignant properties, including CCND1, ERBB2, SNAIL, SLUG, phosphorylated STAT3 (p-STAT3), FAK, FOXM1, ETS1, YAP, HES1, and OCT4, were all significantly downregulated (Figure 4F), an indication of reduction of malignancy in the cancer cells after EZH2 knockdown.

Due to stronger effect of EZH2 knockdown on neuronal differentiation in different cancer cells, we subsequently focused on the role of EZH2 in the regulation of expression of HDAC1, LSD1, or DNMT1, and of β-CAT and SMAD transducers as well.

### EZH2 Is Required for Protein Stability of Its Interaction Partners

**FIGURE 5:**
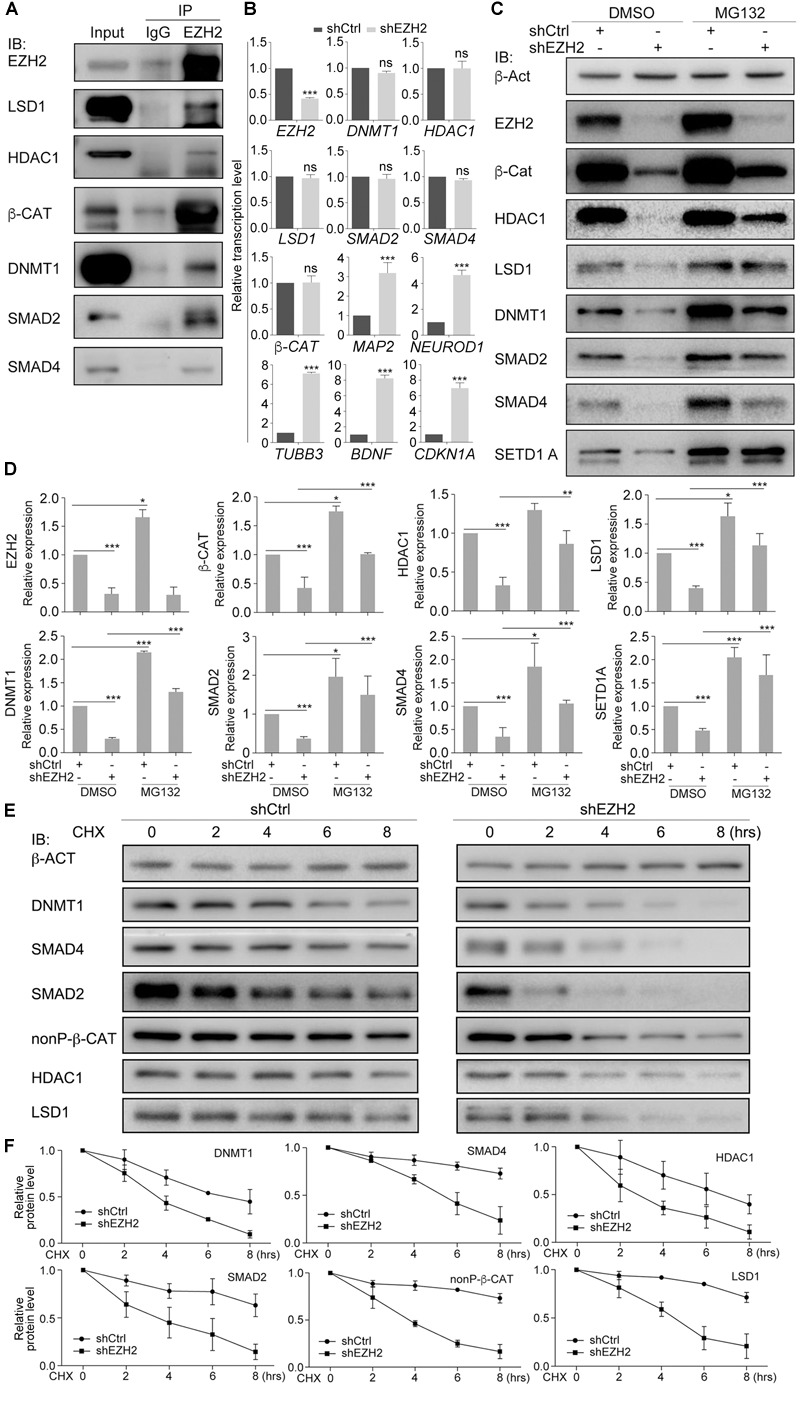
EZH2 interacts with and regulates stability of LSD1, HDAC1, DNMT1, SMAD2/4, and β-CAT in SW480 cells. **(A)** Co-IP shows interactions between EZH2 and other proteins. Co-IP with an IgG antibody was performed in parallel as a negative control, and cellular protein level was used as positive control (Input). **(B)** Regulatory effect of EZH2 knockdown (shEZH2) on gene expression detected with qRT-PCR. Significance in gene expression change was calculated for experiments in triplicate using Student’s *t*-test. Data are shown as mean ± SEM. ns, not significant. ^∗∗∗^*p* < 0.001. **(C)** Effect of EZH2 knockdown on protein level in cells in the absence (DMSO) or presence (MG132) of MG132. **(D)** Quantification of protein levels in **(C)**, significance was calculated for experiments in triplicate using Student’s *t*-test. Quantification is shown as mean ± SEM. ^∗^*p* < 0.05, ^∗∗^*p* < 0.01, ^∗∗∗^*p* < 0.001. **(E)** Analysis of the effect of EZH2 knockdown on protein half-lives by treating HEK293T cells with CHX in a time series. **(F)** Quantification of relative protein levels in triplicate experiments in **(E)**, shown as mean ± SEM.

Co-IP assays showed that EZH2 interacted with HDAC1, LSD1, DNMT1, β-CAT, SMAD2, and SMAD4 (Figure 5A). EZH2 knockdown did not influence gene transcription of these proteins, but caused activation of neuron-specific genes, *MAP2, NEUROD1, TUBB3*, and *BDNF*, as well as *CDKN1A* (Figure 5B), in agreement with IB detection of neuronal protein (Figure 4F) and corroborating again the neuronal differentiation effect. However, EZH2 knockdown caused a severe reduction in HDAC1, LSD1, DNMT1, β-CAT, SMAD2, and SMAD4 (Figures 5C,D), suggesting that EZH2 regulates these proteins at translational level. We also observed a reduction of SETD1A (Figures 5C,D), as observed in TALE treated HepG2 cells. When SW480 cells, both control and knockdown, were treated with the proteasome inhibitor MG132, expression level of these proteins was higher than in cells without MG132 treatment (Figures 5C,D), implying that EZH2 regulation of protein level is dependent on proteasomal activity.

We analyzed the dependence of HDAC1, LSD1, DNMT1, β-CAT, SMAD2, or SMAD4 stability on EZH2 by treating SW480 cells with 50 μM of a protein biosynthesis inhibitor cycloheximide (CHX) with a time series. In control cells, the level of any of the above proteins was decreasing along with increasing time period of CHX treatment (Figures 5E,F), indicating gradual degradation of these proteins in response to inhibition of protein biosynthesis. In contrast, degradation of these proteins seemed to go faster when EZH2 was knocked down, which was also confirmed by quantification (Figures 5E,F). Therefore, EZH2 serves as a regulator of turnover of a battery of key proteins that are involved in both tumorigenesis and neural development.

**FIGURE 6:**
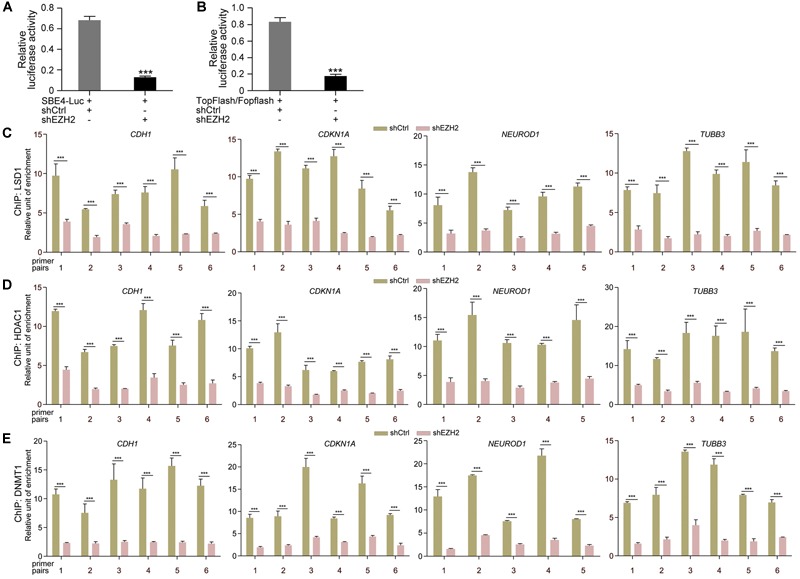
EZH2 knockdown repressed TGFβ and Wnt pathway target gene activation and reduced binding of chromatin modification enzymes to gene promoters. **(A,B)** Effect of EZH2 knockdown on the activity of TGFβ **(A)** or Wnt **(B)** responsive luciferase reporter genes. Relative luciferase activities are shown as mean ± SEM, and significance was calculated for four experiments using Student’s *t*-test. ^∗∗∗^*p* < 0.001. **(C–E)** ChIP assay of binding of LSD1 **(C)**, HDAC1 **(D),** and DNMT1 **(E)** to different promoter regions of neuronal genes in cells without (shCtrl) and with EZH2 knockdown (shEZH2). Relative chromatin binding enrichments are shown as mean ± SEM, and significance was calculated for four experiments using Student’s *t*-test. ^∗∗∗^*p* < 0.001.

Then we tested whether EZH2 regulates transcription of TGFβ and Wnt pathway target genes. Luciferase reporter assays displayed that the activity of TGFβ pathway specific luciferase reporter SBE4-Luc was dramatically reduced in response to EZH2 knockdown (Figure 6A). Similarly, the activity of TopFlash, the Wnt/β-Catenin pathway specific luciferase reporter, was also strongly decreased (Figure 6B). The results indicate that reduced EZH2 leads to instability of SMAD and β-Catenin signal transducers, consequently resulting in repression of target gene transcription of corresponding pathways. Moreover, reduced EZH2 led to significantly reduced binding of LSD1 (Figure 6C), HDAC1 (Figure 6D), and DNMT1 (Figure 6E) to promoter regions of *CDH1, CDKN1A, NEUROD1*, and *TUBB3*, in agreement with increased transcription of these genes in cancer cells after EZH2 knockdown.

### Reduced EZH2 Enhances Ubiquitination of Its Interaction Partners and Increased EZH2 Promotes Expression of These Proteins

**FIGURE 7:**
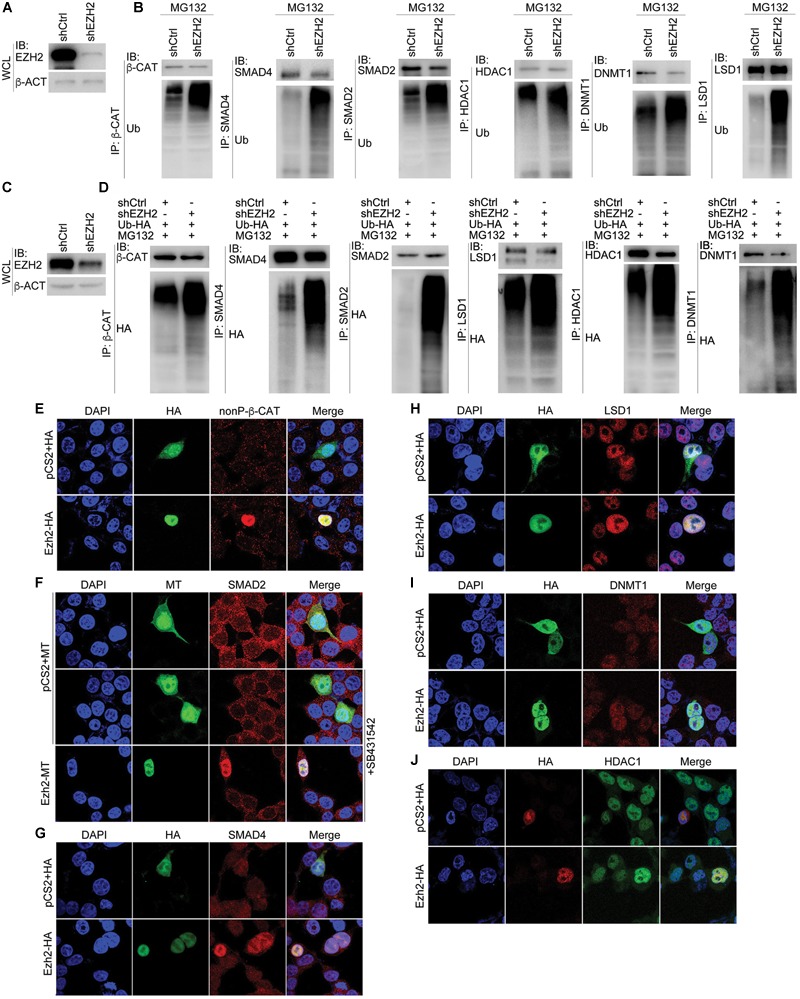
Manipulation of EZH2 level in cells influences ubiquitination and expression of its interaction partners. **(A,B)** In SW480 cells, knockdown of EZH2 increased ubiquitination level on immunoprecipitated proteins (IP) with their respective antibodies. **(A)** shows EZH2 knockdown efficiency in the cells. WCL, whole cell lysate. **(C,D)** In HepG2 cells with ubiquitin overexpression (Ub-HA), EZH2 knockdown increased the level of ubiquitination on proteins that were immunoprecipitated (IP) with antibodies. **(C)** displays EZH2 knockdown efficiency in the cells. WCL: whole cell lysate. **(E–J)** EZH2 overexpression enhances protein level. **(E)** Immunofluorescence (IF) detection of the influence of overexpression of HA-tagged Ezh2 (Ezh2-HA) on expression of nuclear form of ?-CATENIN (nonP-β-CAT). **(F)** IF detection of the effect of overexpression of myc-tagged Ezh2 (Ezh2-MT) on expression of SMAD2 in cells treated with SB431542. **(G–J)** IF detection of the effect of Ezh2-HA overexpression on the expression of SMAD4 **(G)**, LSD1 **(H)**, DNMT1 **(I)**, and HDAC1 **(J)**. In each experiment in **(E–J)**, cells transfected with vector plasmid containing only the HA (pCS2+HA) or myc tags (pCS2+MT) were used as controls. Cell nuclei were visualized with DAPI staining.

We next explored whether EZH2 regulates ubiquitination of its interaction partners. After confirmation of EZH2 knockdown efficiency (Figures 7A,C), β-CAT, SMAD4, SMAD2, HDAC1, DNMT1, and LSD1 were precipitated with their respective antibodies, separately, from control SW480 cells or cells with EZH2 knockdown (Figure 7B). Ubiquitinated protein levels were significantly higher in EZH2 knockdown cells than in control cells (Figure 7B). A similar experiment was also performed with HepG2 cells, in which an HA-tagged ubiquitin was simultaneously overexpressed. After precipitation of each protein with antibody, an HA-tag antibody revealed much stronger ubiquitination of the proteins (Figure 7D). Thus, decrease in EZH2 promotes ubiquitination and consequently proteasomal degradation of these partners.

To further confirm whether EZH2 confers stability of its interaction partners, we overexpressed Ezh2 in HEK293T cells and examined the expression of the other few proteins. Ezh2 overexpression resulted in strong expression of nonphosphorylated, active form of β-Cat (nonP-β-Cat) in cell nucleus, which was barely detectable in the nuclei of cells without Ezh2 overexpression (Figure 7E). SMAD2 was detectable in both cytosol and nuclei of control cells (Figure 7F). Nuclear SMAD2 was significantly reduced in cells treated with a chemical inhibitor of TGFβ receptors, SB435142 (Figure 7F), because it blocked nuclear entry of SMAD2 via inhibition of SMAD2 phosphorylation. Ezh2 overexpression maintained SMAD2 nuclear expression even in the absence of phosphorylation-mediated nuclear translocation (Figure 7F). Moreover, we also observed an elevated expression of SMAD4 (Figure 7G), LSD1 (Figure 7H), DNMT1 (Figure 7I), and HDAC1 (Figure 7J) in cells with overexpressed Ezh2. This assay supports that Ezh2 maintains the expression of its interaction partners.

### EZH2 Confers Protein Stability via Interaction With USP7

**FIGURE 8:**
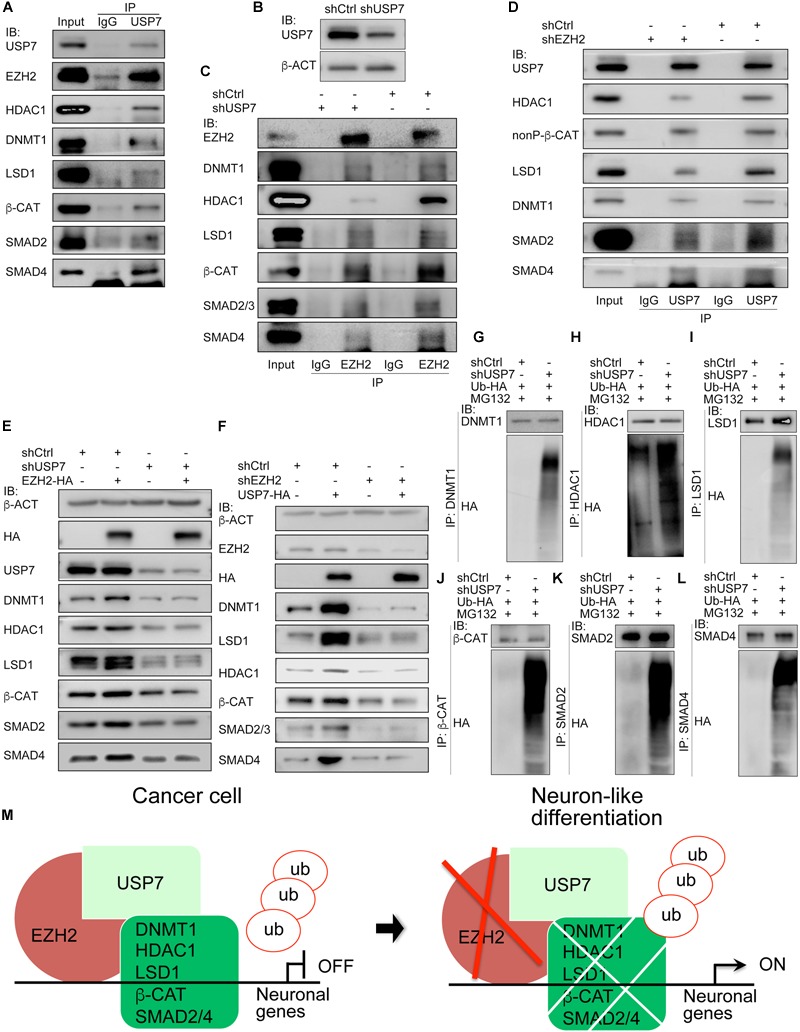
USP7 interacts with EZH2 and is required for EZH2 mediated protein stability. **(A)** Co-IP detection of the interactions between USP7 and EZH2, and between USP7 and EZH2 interaction partners. Immunoprecipitation with IgG was used as a negative control. **(B–D)** The mutual dependence of EZH2 and USP7 on each other’s interaction with proteins detected. **(B)** Detection of knockdown efficiency of a *USP7* short-hairpin RNA (shUSP7). β-ACT was used as a loading control for IB assays. **(C)** Influence of USP7 knockdown on the interactions between EZH2 and other proteins. Proteins were immunoprecicpitated with an EZH2 antibody from cells without and with shUSP7. **(D)** Effect of EZH2 knockdown on the interactions between USP7 and other proteins. Proteins were immunoprecicpitated with a USP7 antibody from cells without and with shEZH2. In **(C,D)**, immunoprecipitation with IgG was used as a negative control. **(E,F)** Interdependence of EZH2 and USP7 on their ability to maintain protein level. **(E)** USP7 knockdown counteracted the enhancement of protein level induced by EZH2 overexpression. **(F)** Knockdown of EZH2 compromised increment of protein level induced by USP7 overexpression. In **(E,F)**, b-ACT expression was detected as a loading control in IB assays. **(G–L)** Effect of USP7 knockdown on the ubiquitination of proteins. Proteins were immunoprecipitated with antibodies against DNMT1 **(G)**, HDAC1 **(H)**, LSD1 **(I)**, β-CAT **(J)**, SMAD2 **(K)**, and SMAD4 **(L)**, respectively, from cells co-transfected with shCtrl or shUSP7 and HA-tagged ubiquitin plasmid, and treated with MG132. Precipitated proteins were detected for their ubiquitination levels using an HA antibody. **(M)** A working model depicting the molecular mechanism by which EZH2 regulates neuron-like differentiation in cancer cells.

We asked why EZH2 can maintain protein stability. As mentioned earlier, a major part of cancer promoting genes were embryonic neural specific, including genes for deubiquitases USP7 and USP39 (Zhang et al., 2017). Co-IP revealed an interaction of EZH2 with USP7. Meanwhile, USP7 also bound to EZH2 interaction partners, i.e., LSD1, HDAC1, DNMT1, SMAD2/4, and β-CAT (Figure 8A). When endogenous USP7 was knocked down with a short-hairpin RNA (shUSP7) (Figure 8B), the EZH2 antibody immunoprecipitated less amount of DNMT1, LSD1, HDAC1, SMAD2/4, or β-CAT protein, as compared to proteins precipitated from cell lysates without USP7 knockdown (Figure 8C). Vice versa, the USP7 antibody precipitated less amount of protein from cell lysates with EZH2 knockdown than from lysates of control cells (Figure 8D). This means that EZH2 and USP7 are interdependent with respect to their ability of interaction with DNMT1, LSD1, etc. The interactions also suggest that USP7 might be involved in EZH2 mediated protein stability. In cells overexpressing Ezh2, expression of LSD1, HDAC1, DNMT1, SMAD2/4, and β-CAT was increased (Figure 8E). When USP7 was knocked down, these proteins were reduced (Figure 8E), similar to the effect of reduced EZH2. However, if Ezh2 was overexpressed together with USP7 knockdown, overexpressed Ezh2 was not able to induce an enhanced expression of these proteins (Figure 8E). Likewise, USP7 overexpression led to upregulation of its interaction partners. When EZH2 was simultaneously knockdown, protein upregulation by USP7 was severely compromised (Figure 8F). Therefore, USP7 and EZH2 are mutually required for their regulation of protein. Furthermore, we observed an enhanced protein ubiquitination in response to reduced USP7 (Figures 8G–L). The results demonstrate that USP7 and EZH2 work together to mediate protein stability. A working model (Figure 8M) summarizes that in cancer cells, EZH2 recruits USP7 and interacts with other proteins, stabilizes these proteins, thereby repressing the expression of neuron-differentiation genes. Reduction in EZH2 protein leads to reduced interaction between USP7 and other proteins, causing increased ubiquitination and degradation of these proteins, consequently resulting in neuronal gene activation.

## Discussion

In the present study, we show that EZH2, LSD1, DNMT1, and HDAC1 form interactions themselves. Meanwhile, they also interact with SMAD proteins and β-CATENIN in cancer cells. These factors regulate cooperatively neuronal gene expression in cancer cells, and likewise, inhibition of these factors in NPCs generates neuronal differentiation. We further show that EZH2 works as a regulator of protein stability, and its presence sustains the expression of key proteins that promote tumorigenesis.

### A Subnetwork That Maintains Undifferentiated State in Cancer Cells and in Neural Progenitor Cells

EZH2, LSD1, DNMT1, and HDAC1 are co-expressed in many types of cancer cells, as well as in neural progenitor/stem cells. We show that these factors work together to restrict neuron gene expression. Synergistic regulatory effect of these factors had been demonstrated for neuronal differentiation in different types of cancer cells (Zhang et al., 2017). The present study shows that the synergistic effect is achieved by forming complexes between these proteins. This does not necessarily mean that these enzymes together form a single complex together at the same time. Interactions between these proteins causes regulatory effects on their protein level since only protein was affected in response to inhibition of the activity of these factors. Downregulated protein and activity essentially reduce their binding to chromatin, subsequently change chromatin modification of the regulated genes, e.g., reduction of H3K27me3 and DNA methylation and increment of H3K27ac and H3K9ac, and consequently result in activation of neuron specific genes or genes inhibiting cell cycle, such as *NEUROD1, TUBB3*, or *CDKN1A*. Hence, EZH2, LSD1, DNMT1, and HDAC1 function together to block the expression of neuron differentiation genes in cells.

Interactions between different epigenetic factors, and between epigenetic factors and signal transducers, particularly SMADs and β-Catenin in the present study, facilitate crosstalk between different types of chromatin modifications and between epigenetic modifications and signal transduction. For instances, functional interplay between HDAC1 and LSD1 causes mutual influences on deacetylation and H3K4 methylation (Lee et al., 2006); LSD1 interacts with DNMT1, so as to maintain global methylation (Wang et al., 2009); DNMT1 forms a complex with β-Catenin, making their expression mutually dependent (Song et al., 2015); DNMT1 interacts with EZH2, forming a transcriptional repressor complex on the promoters of EZH2 target genes (Viré et al., 2006). Since these epigenetic modification enzymes are all specific for neural precursor tissues during neural development (Zhang et al., 2017), the crosstalk enables a concerted regulation of chromatin modifications and signal transduction required for maintenance of undifferentiated state of neural progenitor/stem cells. Loss of the crosstalk will leads to neuronal or neuron-like differentiation from normal neural progenitor/stem cells or cancer cells, respectively.

### EZH2 Maintains a Regulatory Network via Regulation of Protein Stability

One of the key findings of the study is that interactions between these epigenetic modification enzymes may lead to a regulatory effect on protein of their interaction partners. This can be exemplified by the function of EZH2 in maintaining the stability of its interaction partners, including SMAD proteins and β-Catenin. Although the best-known function of EZH2 is to catalyze trimethylation of H3K27, the present study reveals that without EZH2, the proteins to which it binds undergo degradation via the ubiquitin-proteasomal pathway. Correspondingly, higher level of EZH2 enhances protein level of its interaction partners, not only other epigenetic factors, but also signal transducers of TGFβ and Wnt pathways. We propose that EZH2 might function, via bridging deubiquitinase USP7 to target proteins, as a regulator of protein turnover, besides its canonical role in histone methylation. Interaction between EZH2 and USP7 was observed in an *in vitro* assay and a high-throughput study (de Bie et al., 2010; Oliviero et al., 2016). Interestingly, they prefer to interact in undifferentiated NTERA2 carcinoma cells, as compared with NTERA2-derived neuron-like cells (Oliviero et al., 2016). USP7 has been reported to promote the stability of quite a few proteins, especially during cancer initiation and progression (Bhattacharya et al., 2018). β-catenin is stabilized by USP7 mediated deubiquitination (Ma et al., 2014; Novellasdemunt et al., 2017), as also observed in our results.

The fashion of bimodal functions is not unique to EZH2. Indeed, it might be universal for epigenetic modification enzymes. For instances, LSD1 is able to remove methylation of Dnmt1, leading to stabilization of Dnmt1 and maintenance of global DNA methylation (Wang et al., 2009). SETD7 mono-methylates histone H3 at Lys 4. In addition, it stabilizes TP53 and ERα (Chuikov et al., 2004; Subramanian et al., 2008), but destabilizes NF-kB, E2F1, and DNMT1 (Estève et al., 2009; Yang et al., 2009; Kontaki and Talianidis, 2010), via mediating methylation of these proteins. Protein lysine demethylases Kdm2a/b promotes degradation of nuclear β-Catenin after Wnt activation, as a consequence of demethylation (Lu et al., 2015). HDACs are also regulators of protein stability (Choi et al., 2018;Tao et al., 2018).

Another intriguing observation is the regulation of stability of β-Catenin and SMAD transducers by EZH2. Effect of EZH2 on Wnt signal transduction has been described in a few reports. Jung et al. (2013) describes that EZH2 is recruited by PAF to β-Catenin, thereby enhancing Wnt target gene transactivation, independently of methyltransferase activity of EZH2. Another demonstrates that EZH2 knockdown leads to reduced H3K27me3 in the regulated promoter, resulting in transcriptional upregulation of Wnt antagonists (Cheng et al., 2011). Both studies focus on EZH2 regulation of gene transcription. After beginning of our present study shortly, one paper shows that a long noncoding RNA lnc-β-Catm bridges β-Catenin and EZH2, leading to methylation of β-Catenin, suppression of ubiquitination, and consequently, an enhanced β-Catenin stability (Zhu et al., 2016). Therefore, both our study and Zhu et al. (2016) support the role of EZH2 in regulating β-Catenin stability. Wang et al. (2013) reported that SMAD2/4 interacts with EZH2 and displaces EZH2 from the bound locus, leading to altered gene expression. In summary, our results suggest that, once EZH2 is aberrantly upregulated or activated in normal somatic cells due to gain of function mutations or amplifications, a series of cancer-promoting proteins will be abnormally maintained or upregulated, forming a network promoting tumorigenesis. These results explain the key role of EZH2 in tumorigenesis, not only by regulating histone modification, but also by mediating protein stability, thereby regulating other types of chromatin modifications and signal transduction.

### Cancer Cells and Neural Progenitor/Stem Cells Have Similar Differentiation Potential

Inhibition of EZH2, LSD1, HDAC1, and DNMT1, either in combination or individually, stimulates neuron-like differentiation in different cancer cell lines (Zhang et al., 2017; Present study). Together with the similarity of regulatory networks between cancer and neural development, these led us to the proposal that cancer regulatory signals confer cancer cells with properties of neural progenitor/stem cells (Cao, 2017; Zhang et al., 2017). Besides localized expression in neural precursor/progenitor cells during embryonic neural development (Zhang et al., 2017), functional studies show that EZH2 is expressed in and maintains proliferation of NPCs (Zhang et al., 2014) and a decrease in EZH2 enhances neuron differentiation (Yu et al., 2013); besides, Ezh2 is able to dedifferentiate astrocytes into neural stem cells (Sher et al., 2011). These studies all support the ability of EZH2 to maintain or confer neural stemness in cells. Inhibition of USP7 causes neuron differentiation in neural peogenitor/stem cells, leads to activation of neuronal genes and repression of neural stem marker genes, suggesting a requirement of USP7 for maintenance of neural stemness (Huang et al., 2011; Nicklas et al., 2019). β-Catenin is also essential for the maintenance of proliferation of neural progenitors (Zechner et al., 2003); knockdown of Smad2 enhances neurogenesis (Míguez et al., 2013). Moreover, HDAC1/2 and LSD1 expression is decreased during neuronal differentiation (Sáez et al., 2015), suggesting that lower HDAC and LSD1 activities are required for neuronal differentiation. Actually, destabilization of LSD1 promotes neurogenesis (Han et al., 2014) and HDAC inhibition induces neuronal differentiation via activation of NeuroD1 (Hsieh et al., 2004). In addition, DNMT1 and EZH2 interact together to repress transcription of neuron specific gene *MYT1* (Viré et al., 2006). Therefore, these epigenetic modification enzymes and signal transducers serve to maintain undifferentiated state of neural progenitor/stem cells. Their activities should be repressed in order for neuronal differentiation. This is the very effect we observed for neuronal or neuron-like differentiation from NPCs and cancer cells. This reinforces our proposal above, and implies again that the main cancer regulatory network also functions in the similar way in neural progenitor/stem cells. Besides inhibition of epigenetic factors, blocking of PTBP1 (or PTB) in tumor cell lines caused neuronal differentiation (Xue et al., 2013), supporting again the intrinsic association between cancer cells and neural progenitor/stem cells. The study itself did not deal with the role of PTBP1 in cancer. In fact, it promotes various types of cancer, e.g., pancreatic and breast cancer (He et al., 2014; Calabretta et al., 2016). Expectedly, *ptbp1* expression is localized to embryonic neural precursor tissues (Noiret et al., 2012). Besides cancer cells, cancer tissues also show neural differentiation effect (Zhang et al., 2010). Cancer immunotherapy seems to progress quickly. However, immunotherapy resistance also occurs. Factors conferring immunotherapy resistance, such as PBRM1 (Pan et al., 2018), β-Catenin (Spranger et al., 2015) or LSD1 (Sheng et al., 2018), etc., are embryonic neural specific (Zhang et al., 2017). CGAS and CHAF1B are known only recently for their roles in promoting tumorigenesis (Liu et al., 2018; Volk et al., 2018). Again, *Cgas* is only expressed in nervous system in mouse, as known from mouse database^2^, and *chaf1b* is localized also to nervous system in zebrafish embryos (Fischer et al., 2007). There are numerous additional reports describing the relationship between neural factors and their roles in cancer promotion (Cao, 2017; Zhang et al., 2017). In summary, our previous (Cao, 2017; Zhang et al., 2017) and present studies establish an intrinsic association between cancer cells and a specific cell type, the neural progenitor/stem cells. The association is not merely based on the neuronal differentiation phenotype in cancer cells, but on a combination of data comprised of neuronal phenotype in cancer cells, the specific or enriched expression of hundreds (not just a few) of cancer promoting genes (including *EZH2* and a few others in the present study) in normal neural progenitor/stem cells, the functions of these cancer genes in normal neuronal differentiation, as well as implications from other *in vivo* and *in vitro* cancer studies (e.g., those cited in the text). The similarity between cancer cells and neural progenitor/stem cells provides not only an insight into the essence and unified framework for cancer initiation and progression, but also alternative options for novel strategies for cancer therapy, such as by cell differentiation/transdifferentiation effects.

## Author Contributions

YC and AL conceived the research. AL, MZ, and LX performed cell assays. AL, LC, MZ, ZZ, and NC performed biochemical studies. AL, LC, and XY performed molecular biology experiments. All authors analyzed the data. YC wrote the manuscript.

## Conflict of Interest Statement

The authors declare that the research was conducted in the absence of any commercial or financial relationships that could be construed as a potential conflict of interest.

## Footnotes

1 http://www.ncbi.nlm.nih.gov/geo/query/acc.cgi?acc=GSE118593

2 http://www.informatics.jax.org/
