# Supplementary material for: EZH2 Regulates Protein Stability via Recruiting USP7 to Mediate Neuronal Gene Expression in Cancer Cells
Source: Front Genet. 2019 May 3;10:422. doi: 10.3389/fgene.2019.00422 (PMC6510286; doi:10.3389/fgene.2019.00422)
Supplement: Supplementary file 1 [file Table_1.DOCX]

**Supplementary Table 1**. Primers for ChIP-qPCR. Predicted amplified regions are indicated by numbers at the end of each reverse primer. The first base of the transcriptional start site is designated as +1.

| Primers | 5’-3’ |
| --- | --- |
| ChIP-hCDH1-F1 | AGAGGGGCATCCGTAGAAAT |
| ChIP-hCDH1-R1 | CACCGTTCCCTTTCAGTCTC (558/767) |
| ChIP-hCDH1-F2 | TAGAGGGTCACCGCGTCTAT |
| ChIP-hCDH1-R2 | TCACAGGTGCTTTGCAGTTC (-170/30) |
| ChIP-hCDH1-F3 | ATGGCTCACACCTGAAATCC |
| ChIP-hCDH1-R3 | AGTACAGGTGCACACCACCA (-649/-456) |
| ChIP-hCDH1-F4 | ATCAGCCTCGGCAACATAGT |
| ChIP-hCDH1-R4 | TACAGACGGGGTTTCACCAT (-1052/-869) |
| ChIP-hCDH1-F5 | GCCAGGATGGTCTCAATCTC |
| ChIP-hCDH1-R5 | CCCTATGCTGTTGTGGGACT (-1434/-1243) |
| ChIP-hCDH1-F6 | GCAGTGAGCCAAGAACACAC |
| ChIP-hCDH1-R6 | GAGATCGCGCCACTGTACTC (-1790/-1573) |
| ChIP-hCDKN1A-F1 | CTTCAAGGCAGTGGGAGAAG |
| ChIP-hCDKN1A-R1 | GATTGTGGCTAAACCCCAGA (553/711) |
| ChIP-hCDKN1A-F2 | AGGAAGGGGATGGTAGGAGA |
| ChIP-hCDKN1A-R2 | CTCCCAGCACACACTCACAC (39/188) |
| ChIP-hCDKN1A-F3 | GAGGCAGAATTGCTTGAACC |
| ChIP-hCDKN1A-R3 | ATAGGGGCAGTCAGCTTTCA (-566/-339) |
| ChIP-hCDKN1A-F4 | TCTCAGCTCACTGCAACCTC |
| ChIP-hCDKN1A-R4 | TGGTGGCTTACGCCTGTAAT (-985/-761) |
| ChIP-hCDKN1A-F5 | GCAGGTGTGATGACCAACAA |
| ChIP-hCDKN1A-R5 | TTTCATCCATTCATTCAAAAACC (-1331/-1139) |
| ChIP-hCDKN1A-F6 | CTGAGGGGAGGCTCATACTG |
| ChIP-hCDKN1A-R6 | AGAGAGGCATCCTCCAGACA (-1884/-1475) |
| ChIP-hNEUROD1-F1 | TTTTACGCACATTGGGAGTG |
| ChIP-hNEUROD1-R1 | AGCGGTAACAGGTAGCAGGA (627/793) |
| ChIP-hNEUROD1-F2 | AGGCCACTCGCTCTGATCTA |
| ChIP-hNEUROD1-R2 | CCTTTGTGGCCAGAAGAAAG (-367/-184) |
| ChIP-hNEUROD1-F3 | AGCTCGCTTTGAGGACAAGA |
| ChIP-hNEUROD1-R3 | ATCGTCCTCTCCCAGTTCCT (-843/-676) |
| ChIP-hNEUROD1-F4 | GAGTTCGCAGCCATTAATCC |
| ChIP-hNEUROD1-R4 | TAGTCCAGGCATTGACCACA (-1248/-1034) |
| ChIP-hNEUROD1-F5 | CTGGCTAGGACCCTCTTCCT |
| ChIP-hNEUROD1-R5 | AGACCTGAAACCCTCTGCAA (-1669/-1490) |
| ChIP-hTUBB3-F1 | CCGACGCTTTGTTTCTTCTC |
| ChIP-hTUBB3-R1 | GGCTTTGTACGGAGGGTCTT (802/975) |
| ChIP-hTUBB3-F2 | GGGGTTCGTCTGTACATCGT |
| ChIP-hTUBB3-R2 | TCAGAGAAGGAAGGGAGCAA (291/450) |
| ChIP-hTUBB3-F3 | TCTCGCTGAAGAGACCACCT |
| ChIP-hTUBB3-R3 | AGGAAGCAGCTCCCAGTTCT (-318/-138) |
| ChIP-hTUBB3-F4 | AAACCCCAGCCTAGAGGAAG |
| ChIP-hTUBB3-R4 | CAGGCATCTCAGAGTGGACA (-811/-595) |
| ChIP-hTUBB3-F5 | CCTAGTGGTATGGGGCTGAG |
| ChIP-hTUBB3-R5 | TTTCTGGAAGGGGCTAAGGT (-1224/-1017) |
| ChIP-hTUBB3-F6 | CTCCAGGCAGGACTTCTCAC |
| ChIP-hTUBB3-R6 | GATGCTGCTCAGTCATTGGA (-1607/-1444) |
