# Supplementary material for: EZH2 Regulates Protein Stability via Recruiting USP7 to Mediate Neuronal Gene Expression in Cancer Cells
Source: Front Genet. 2019 May 3;10:422. doi: 10.3389/fgene.2019.00422 (PMC6510286; doi:10.3389/fgene.2019.00422)
Supplement: Supplementary file 2 [file Table_2.DOCX]

**Supplementary Table 2**. Primers for RT-qPCR

| Gene | Primers (5’-3’) |
| --- | --- |
| *β-CAT* | Forward primer: TCCCACTAATGTCCAGCGTT |
|  | Reverse primer: ATGGACCATAACTGCAGCCT |
| *BDNF* | Forward primer: GCAAACATCCGAGGACAAGG |
|  | Reverse primer: CACCCTGGACGTGTACAAGT |
| *CCND1* | Forward primer: GCATGTTCGTGGCCTCTAAG |
|  | Reverse primer: CGTGTTTGCGGATGATCTGT |
| *CDH1* | Forward primer: CGGACGATGATGTGAACACC |
|  | Reverse primer: TTGCTGTTGTGCTTAACCCC |
| *CDKN1A* | Forward primer: CACTCGTCAAATCCTCCCCTT |
|  | Reverse primer: TCCAGTGGTGTCTCGGTGA |
| *DNMT1* | Forward primer: GAGCCACAGATGCTGACAAA |
|  | Reverse primer: TGCCATTAACACCACCTTCA |
| *EZH2* | Forward primer: CCGCTGAGGATGTGGATACT |
|  | Reverse primer: CTTGGTGTTGCACTGTGCTT |
| *GAPDH* | Forward primer: GTCAGTGGTGGACCTGACCT |
|  | Reverse primer: CCCTGTTGCTGTAGCCAAAT |
| *HDAC1* | Forward primer: ATATCGTCTTGGCCATCCTG |
|  | Reverse primer: GGCTTGAAAATGGCCTCATA |
| *LSD1* | Forward primer: ATCTGCAGTCCAAAGGATGG |
|  | Reverse primer: GCCAACAATCACATCGTCAC |
| *NEUROD1* | Forward primer: GAGACGCATGAAGGCTAACG |
|  | Reverse primer: AGTCCGAGGATTGAGTTGCA |
| *NF-L* | Forward primer: AGACCCTGGAAATCGAAGCA |
|  | Reverse primer: TCGCCTTCCAAGAGTTTCCT |
| *NF-M* | Forward primer: AAATGGAAGAGGCCCTGACA |
|  | Reverse primer: TCTTCGGCTTGGTCTGACTT |
| *SMAD2* | Forward primer: CTTTGTGCAGAGCCCCAATT |
|  | Reverse primer: CTTGTTACCGTCTGCCTTCG |
| *SMAD3* | Forward primer: GCAGAACGTCAACACCAAGT |
|  | Reverse primer: CGAACTCACACAGCTCCATG |
| *SMAD4* | Forward primer: CAGATAGCATCAGGGCCTCA |
|  | Reverse primer: TGGAAATGGGAGGCTGGAAT |
| *TUBB3* | Forward primer: TGGACATCTCTTCAGGCCTG |
|  | Reverse primer: TTCATGATGCGGTCGGGATA |
